# Rehabilitation and Recovery in a Patient With Cerebellar Atrophy and Ataxia Following Femoral Fracture: A Case Report

**DOI:** 10.1155/cro/9183409

**Published:** 2026-05-03

**Authors:** Morteza Gholipour, Mahdi Mohammaditabar, Fatemeh Abbasi

**Affiliations:** ^1^ Clinical Research Development Unit, Akhtar Hospital, Shahid Beheshti University of Medical Sciences, Tehran, Iran, sbmu.ac.ir; ^2^ Bone, Joint and Related Tissues Research Center, Akhtar Orthopedic Hospital, Shahid Beheshti University of Medical Sciences, Tehran, Iran, sbmu.ac.ir; ^3^ Department of Medicine, To.C., Islamic Azad University, Tonekabon, Iran, azad.ac.ir

**Keywords:** ataxia, cerebellar atrophy, femur fracture, recovery, rehabilitation

## Abstract

Patients with neurological conditions like cerebellar atrophy and ataxia face many challenges when recovering from fractures. This case report details the management and recovery of a 45‐year‐old woman with severe, pre‐existing cerebellar atrophy and ataxia who sustained a right femoral shaft fracture after a fall. Before her injury, her neurological impairment required her to use a walker to move around. She underwent open reduction and internal fixation (ORIF) and was given a 3‐month period during which she could not put weight on her leg. Her rehabilitation involved a team approach focused on specific goals, emphasizing early knee range‐of‐motion exercises. Given her dual diagnosis, the intensive program focused on various training components, including progressive resistance, coordination drills, and challenging balance exercises. These exercises have been shown to reduce ataxia symptoms and improve motor function significantly. The tailored intervention operated on the idea that the quality and difficulty of rehabilitation are key factors for positive outcomes in patients with degenerative cerebellar ataxias (DCAs). After 3 months, the fracture healed well, and the patient moved to full weight‐bearing, showing significant improvements in mobility and independence. This case highlights that a unified, specialized, and evidence‐based team approach can lead to successful functional recovery, even in complex neuro‐orthopedic situations. It aligns with modern rehabilitation methods that focus on enhancing the quality of life by reducing secondary impairments.

## 1. Introduction

Managing orthopedic injuries in patients with significant neurological impairments presents unique challenges that require a comprehensive, multidisciplinary approach. Neurological conditions such as cerebellar atrophy and ataxia significantly impact balance, coordination, and mobility, increasing the risk of falls and subsequent fractures [1]. The interplay between neurological deficits and musculoskeletal injuries necessitates tailored rehabilitation strategies to optimize functional recovery [2].

Effective management of such cases involves collaboration among orthopedic surgeons, neurologists, and physiotherapists to address both the mechanical aspects of the injury and the underlying neurological condition [3]. Early initiation of rehabilitation, with careful monitoring, is crucial to prevent complications such as joint stiffness and muscle atrophy while accommodating the patient′s neurological limitations [4].

Patient compliance with rehabilitation protocols significantly predicts successful outcomes [3]. Customized treatment plans that consider the individual′s neurological status, functional goals, and potential barriers to adherence are essential [5]. This case highlights the importance of a multidisciplinary approach in managing orthopedic injuries in patients with neurological impairments, demonstrating that favorable outcomes are achievable through coordinated care and personalized rehabilitation strategies.

## 2. Case Presentation

**Figure 1:**
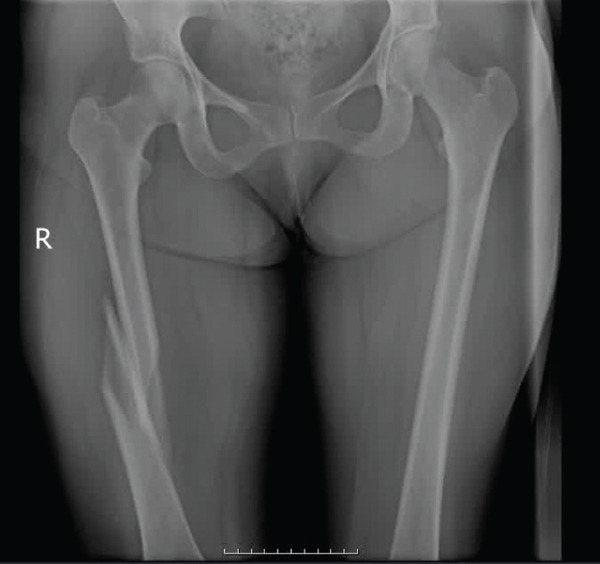
Patients x‐ray, right femoral shaft fracture.

A 45‐year‐old female with a history of advanced cerebellar atrophy and ataxia due to the involvement of the spinocerebellar arteries was brought to our institute following a fall in her house (Figure 1). She had been dependent on a walker for ambulation because of her neurologic disabilities. She had a right femoral shaft fracture on examination. Because of the nature and severity of the injury, the fracture was internally fixed with a plate (Figure 1).

Postoperatively, the patient was maintained on a nonweight‐bearing status for 3 months to allow the fracture to heal completely. Early rehabilitation was started within 2 weeks of the surgery with knee range‐of‐motion exercises to avoid joint stiffness and to maintain functional ability [6]. The rehabilitation was carefully monitored because of the ataxia and balance problems of the patient. (Table 1) briefly explains our rehabilitation program.

**Table 1:** The key components of the multiphase rehabilitation protocol were as follows (adapted from intensive DCA rehabilitation models).

Intervention phase	Primary goals	Specific therapeutic interventions	Intensity/duration
Phase 1: Early postoperative	Maintain ROM; prevent stiffness/atrophy; preparatory strengthening (nonoperative side and trunk).	Active and passive knee ROM exercises. General conditioning. Upper limb strengthening with light or manual resistance. Lower limb exercises for the unaffected leg.	Started 2 weeks postsurgery.
Phase 2: Strength and functional mobility	Improve generalized weakness; enhance core stability and functional capacity (e.g., bed mobility, transfers).	**Progressive Resistance Training (PRT)** for upper and lower limbs (e.g., utilizing weight cuffs or manual resistance, progressively increasing intensity based on tolerance). **Task-specific training** for ADLs, such as **sit-to-stand training**.	Intensive therapy, focusing on strength, potentially 10 repetitions per set.
Phase 3: Balance, coordination and gait	Achieve independent and safer walking; improve dynamic balance and coordination.	**Coordination drills** (e.g., Frenkel′s exercises). **Balance exercises** (e.g., balance board activities, single‐leg stance, gradually increasing the duration). **Gait training:** walking parallel to bars, **walking in front of a mirror** (for visual feedback). **Stair climbing** (e.g., up and down a stepper, performed multiple repetitions).	Highly customized intensity, focusing on challenging balance.

**Figure 2:**
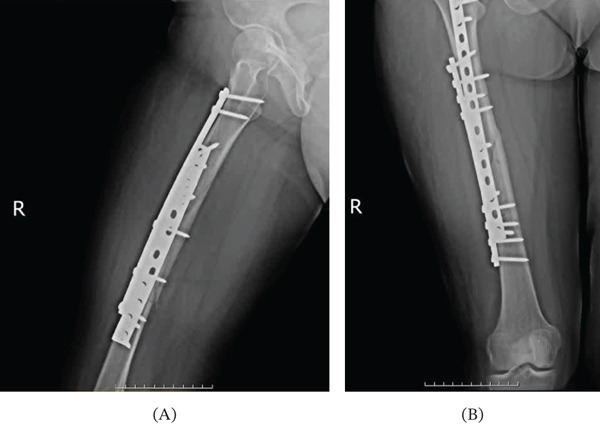
Follow‐up x‐ray after 3 months, revealing good healing of the fracture.

At the 3‐month follow‐up, both the clinical and radiological examination revealed good healing of the fracture (Figure 2). The patient continued physiotherapy to strengthen the weak limb and improve mobility further, and gradually progressed to full weight‐bearing activity. Despite her neurological condition, the patient was exceptionally compliant with the rehabilitation regimen, which proved to be one of the prime predictors of good functional results.

This case emphasizes the intricate challenges associated with the management of orthopedic injuries in individuals who exhibit significant neurological impairments, thereby underscoring the critical importance of a collaborative, multidisciplinary strategy. Successful cooperation between orthopedic surgeons, neurologists, and physiotherapists was determined to be crucial for the patient′s recovery [7]. The patient′s experience demonstrates that favorable results may be achieved in complicated scenarios through customized treatment plans and rehabilitation techniques.

## 3. Discussion

The care for this 45‐year‐old woman with severe cerebellar atrophy and ataxia after an ORIF for a femoral shaft fracture shows the complex problems linked to neuro‐orthopedic injuries [8, 9]. The usual orthopedic treatment, which includes ORIF and nonweight‐bearing status, had to be carefully combined with a specialized neuro‐rehabilitation plan. This plan addressed the difficulties caused by her cerebellar issues, including poor balance and coordination [10].

The success seen in this case strongly matches current evidence that supports intensive, multifaceted physiotherapy for CA patients [11].

Systematic reviews show that physiotherapy, using multifaceted training programs that include muscle strengthening, coordination training, gait training, and activities of daily living training, significantly reduces ataxia symptoms. The pooled mean difference in SARA scores is −1.41 [11]. Additionally, balance training and aerobic exercise, such as cycling, also have a significant impact on reducing ataxia symptoms.

This approach focused on personalized training, recognizing that people with ataxia deal with issues like weaker anticipatory postural control and coordination problems. This customized method is backed by the idea that creating a home‐based exercise program that provides enough challenge to balance is the best predictor of positive results in individuals with cerebellar ataxia [10].

The goal‐oriented protocol successfully improved the patient′s functional outcomes. This aligns with findings that coordination training enhances motor function and helps patients reach important personal goals, such as walking independently.

The patient′s strong adherence was noted as a key predictor of good functional results. Large‐scale studies on degenerative cerebellar ataxias clarify the link between rehabilitation components and overall quality of life [12].

Research using structural equation modeling in DCA populations has shown that quality of life is directly affected by activity limitations, secondary impairments like weakness, rigidity, and fatigue, and the quality of rehabilitation [12].

The amount of rehabilitation—such as the duration, variety, and timing of therapies—indirectly impacts quality of life mainly by reducing secondary impairments. Importantly, the quality of rehabilitation, which reflects patient engagement, motivation, and perceived effectiveness, has a direct and independent effect on quality of life [12]. Thus, the patient′s exceptional compliance indicates a high quality of rehabilitation, contributing to her outstanding functional results despite her complex dual diagnosis.

### 3.1. Considering Advanced Neurorehabilitation

For patients who have difficulty achieving better functional balance, often needed for full independence, VR‐guided balance training is becoming a practical therapy option [13]. VR systems, such as mediVR KAGURA, offer intensive, repetitive, task‐oriented training with multisensory feedback. This feedback is essential for promoting neuroplasticity [14].

VR systems address the common issue of adjusting task difficulty. They let users customize the speed, location, and number of objects to suit the patient′s abilities [13]. This technology gives direct, accurate, multisensory feedback through sight, sound, and touch. For cerebellar patients, who show less feedforward control and depend on outside feedback to fix mistakes, this direct sensory input is believed to enhance important feedforward learning more effectively than traditional methods [14].

Innovative immersive systems like the Computer Assisted Rehabilitation ENvironment (CAREN) have a 6‐degree‐of‐freedom platform along with a VR screen [14]. A pilot study using CAREN in patients with cerebellar ataxia showed improvements in certain gait parameters, including better trunk rotation, stability, coordination, and a reduced risk of falling. Improving trunk rotation is vital because a lack of coordination between the upper and lower body can lead to an unstable, wide‐based gait in these patients.

These contemporary methods highlight the significance of intensive, customized rehabilitation tools—both traditional and technological—that follow the proven principles employed in this patient′s recovery. The successful outcome underscores the necessity of a cohesive, multidisciplinary approach focused on personalized recovery goals in complex neuro‐orthopedic cases. Unfortunately, despite impressive results on paper, such rehabilitation programs are not yet widely accessible.

## 4. Conclusion

The successful achievement of positive functional outcomes, even with serious neurological impairment, highlights the need for a special, personalized, team‐based neuro‐physiotherapy plan. This method matches current evidence that supports thorough and challenging rehabilitation programs in various areas.

## Funding

No funding was received for this manuscript.

## Conflicts of Interest

The authors declare no conflicts of interest.

## Data Availability

The data that support the findings of this study are available from the corresponding author upon reasonable request.
